# Process, Design and Materials for Unidirectionally Tilted Polymeric Micro/Nanohairs and Their Adhesion Characteristics

**DOI:** 10.3390/polym8090326

**Published:** 2016-09-02

**Authors:** Hyeon Seong Im, Jong Uk Kim, Sungwon Han, Tae-il Kim

**Affiliations:** 1 Center for Neuroscience Imaging Research (CNIR), Institute of Basic Science, Suwon 440-746, Korea; skin119@naver.com (H.S.I.); kjo1687@naver.com (J.U.K.); 2 School of Chemical Engineering, Sungkyunkwan University (SKKU), Suwon 440-746, Korea; 3 Department of Chemistry, Sungkyunkwan University (SKKU), Suwon 440-746, Korea; 1398@skku.edu

**Keywords:** gecko-like dry adhesive, high aspect ratio polymeric nanostructure, unidirectionally tilted structures, adhesiveless transfer printing, climbing robotics

## Abstract

Recent research in the field of gecko-inspired dry adhesive has focused on modifying the material and structural properties of polymer-based nanohairs. Polymers such as polystyrene (PS), high-density polyethylene (HDPE), ultraviolet curable epoxy (SU-8), polyurethane acrylate (PUA), polycarbonate (PC), and polydimethyl siloxane (PDMS) can fulfill many mechanical property requirements, are easily tunable, and can be produced via large-scale fabrication. However, the fabrication process for tilted structure remains challenging. The tilted structure is a crucial factor in high-degree conformal contact, which facilitates high adhesion, low effective modulus, and directional adhesion properties. Recent studies have attempted to create a tilted structure by applying beam irradiation, mechanical and thermal stress, and magnetic fields. This review provides a comprehensive investigation into advanced strategies for producing tilted polymeric nanostructures and their potential applications in the near future.

## 1. Introduction

Synthetic gecko dry adhesives are regarded as a promising alternative for application in commercial viscoelastic adhesives, medical patches, transfer printing, and climbing robots due to their remarkable properties such as relatively high adhesion, self-cleaning, and durability [1,2,3,4,5,6,7]. These adhesive systems were inspired by the setae on a gecko’s feet, which have a complicated arrangement consisting of a hierarchical hairy structure containing a number of micrometer-sized setae and nanometer-sized spatulae composed of a stiff material, β-keratin, with an elastic modulus of ~2 GPa [8,9,10,11,12]. In nature, geckos can climb various surfaces regardless of their roughness, even vertical walls. It is particularly important that a gecko’s setae are tilted in form, not straight, such as those of a lotus or insects [8,13,14]. When these structures contact a substrate, the tilted high aspect ratio structures smoothly bend and significantly increase the contact area, which maximizes the van der Waals force, and they even show a different adhesion force depending on the direction of attachment and detachment [15,16,17,18]. This enables geckos to move quickly. The other advantage of the structure is reusability in that its adhesion does not decrease even with repeated contact, since the fine structure produces a self-cleaning effect and the stiffness of the structure allows it to endure external forces [19,20,21,22].

Many investigations of polymeric materials such as polystyrene (PS), high-density polyethylene (HDPE), ultraviolet curable epoxy (SU-8), polyurethane acrylate (PUA), polycarbonate (PC), and carbon nanotubes (CNTs) have sought to replicate this structure [23,24,25,26,27,28,29,30]. The CNT-based product can be manufactured with a fine structure at the nanometer level and provides a high degree of mechanical strength and adhesion force. However, in the manufacturing process, high-cost steps are required such as a high temperature and a high vacuum state, and large-scale production is difficult. These limitations have encouraged engineers to fabricate synthetic gecko dry adhesive primarily using polymeric materials. Polymer-based adhesive is suitable for large-scale production, as the process is not time-consuming, involving just molding and curing processes, and the materials are inexpensive. In addition, the mechanical properties of a polymeric material-based structure can be controlled [31,32].

This paper provides a summary of synthetic dry adhesives, especially polymer-based tilted micro/nanohairs. Previous reviews comprehensively described fabrication processes, designs, and adhesion characteristics of synthetic dry adhesives; however, they did not focus on tilted structures [33,34,35,36]. The manufacturing processes involved in tilted structures are generally more sophisticated, requiring additional steps and molding methods. Thus, we describe these processes in detail.

## 2. The Effect of the Tilted Hairy Nanostructure of Gecko Feet

**Figure 1:**
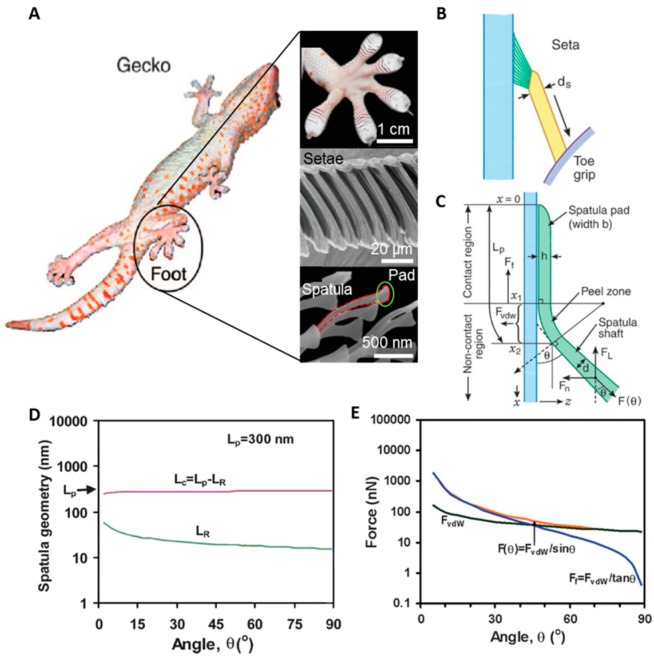
Gecko foot structure and its analysis. (**A**) The hierarchical structures of a gecko’s foot, which consist of setae, spatulae, and pads. (Foot image reproduced from [11], with permission from Annual Reviews); (**B**,**C**) Schematic diagram of the setae and parameters related to adhesion; (**D**,**E**) van der Waals force adhesion of the setae calculated using different contact angles with the substrate. (Figure reproduced from [10], with permission from the National Academy of Science, Washington, DC, USA.)

In natural systems, many living things, such as beetles, flies, spiders, and geckos, show a wide range of adhesion characteristics [8]. Gecko hair is a popular research topic because of its unique properties. Its structure enables conformal adhesion regardless of the type of surface; this differs from other adhesion systems such as simple rakes, micrometer-sized monolayer setae, and wet adhesion [8,37], which provide limited adhesion. What makes this intimate contact possible is that the hair is unidirectionally tilted. When contact occurs, the hair is easily deformed and contacts a wider area than vertical types of hair. As can be seen in Figure 1, gecko hair consists of setae, which are 120 μm long and have a diameter of 4.2 μm, and spatulae, which have a length of 800 nm and a diameter of 100 nm, corresponding to an aspect ratio of about ~30 [10]. An analysis of this structure’s adhesion is presented in Figure 1B–E. The pulling forces, *F*_θ_, are determined by the normal adhesion force, *F*_vdW_ (from *x*_1_ to *x*_2_), and the lateral friction force, *F*_f_ (from *x*_0_ to *x*_1_). The plots in Figure 1D–E illustrate the change of the force contribution with varying angle, θ, which indicates the value between the surface and structure. The contact length, *L*_R_, increases with decreasing angle, such that the normal adhesion force, *F*_vdW_, increases. The friction force also increases nearly two orders of magnitude with decreasing angle. Accordingly, the angle determines the pulling force, *F*_θ_.

## 3. Various Fabrication Methods for the Tilted Nanohairs

Most research concerning artificial gecko dry adhesive has focused on utilizing polymeric materials, for which a simple manufacturing process can be used to create various angles, tips, and a hierarchical structure as well as tunable properties including surface energy and modulus. Here, we highlight the fabrication methods and divide them into two categories, depending on the fabrication methods. The first method, the molding technique, requires no additional treatment to prepare an angled master mold. The second method involves irradiating a beam, inducing thermal shrinkage, applying a direct physical force like rolling, and utilizing magnetism.

### 3.1. Molding Method

**Figure 2:**
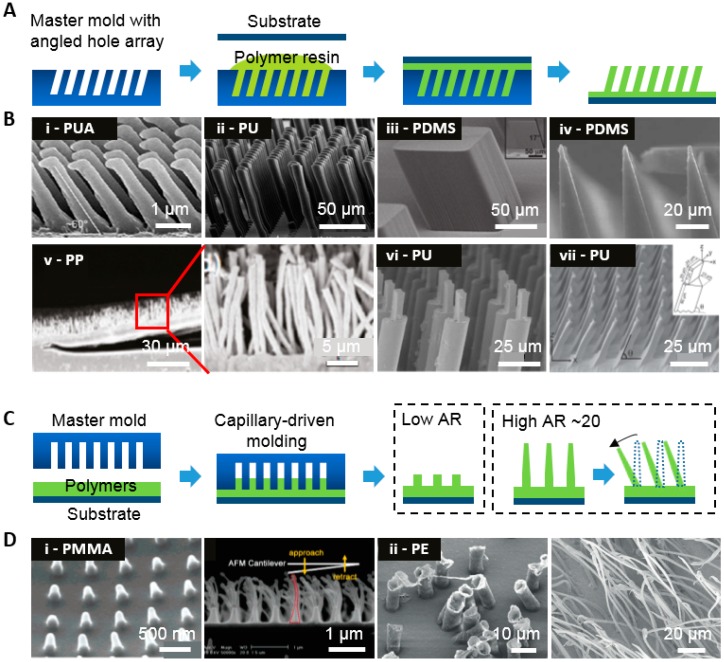
Schematic diagram and representative SEM images of the molding technique for fabricating tilted hairs. (**A**) Micro-/nanohairs obtained via soft molding on tilted slanted etched holes in the master mold; (**B**) various tilted hairs with (i) PUA, (ii,vi,vii) PU, (iii–iv) PDMS, and (v) PP. ((i) reproduced from [41], with permission from the National Academy of Science, Washington, DC, USA; (ii) reproduced from [42], with permission from the American Chemical Society; (iii) reproduced from [4], with permission from John Wiley & Sons; (iv) reproduced from [43], with permission from the Royal Society & Chemistry; (v) reproduced from [44], with permission from the American Chemical Society; (vi) reproduced from [45], with permission from the American Chemical Society; (vii) reproduced from [46], with permission from John Wiley & Sons); (**C**) Drawing of vertical polymeric hairs above the glass transition temperature by controlled adhesion between the polymer and mold; (**D**) (i) PMMA nanohairs and (ii) PE micro hairs obtained via nanodrawing methods. ((i) reproduced from [23], with permission from the American Chemical Society; (ii) reproduced from [47], with permission from IOP publishing Ltd.).

Molding can be easily adapted to a micro/nanoscale fabrication process. Due to its cost efficiency and scalability, it is suitable for mass production by roll-to-roll manufacturing. The micro/nano angled hairs are cast by various polymer precursors and cured by UV exposure or thermal curing (Figure 2). In general, the molds with micro/nano hole arrays are prepared by photolithography, where tilted exposures are required under a patterned shadow mask to create an angled structure. Here, the desired angle can be calculated by Snell’s law and by reactive ion etching (RIE), in which the holder should be tilted to the certain angle followed by subsequent etching [38,39,40].

Figure 2B(i–iv) show representative SEM images of polymeric hairs produced using the molding technique. PDMS and PUA are widely used because of their tunable characteristics. In Figure 2B(i), nanohair structures with a tip diameter of 600 nm, bottom diameter of 700 nm, and a height of 3 μm are obtained using PUA cured by UV exposure after being spun on a negative master mold [41]. Here, the developed methods are chosen to produce the master mold, which is fabricated by an RIE set with a Faraday cage, allowing for control of the etched angle. With conventional RIE, angle control is difficult. SU-8 based angled hair with a diameter of 25 μm, a height of 75 μm, and an angle of 25° was created by photolithography (Figure 2B(ii)) [42]. UV was exposed to an SU-8 layer on a tilted wafer with an angle of 45°, which formed hairs with an angle of 75°. PDMS-based tilted hairs with diameters of 20 and 100 μm, and heights of 80 and 100 μm were also fabricated using the same methods, respectively (Figure 2C(iii,iv)) [4,43]. A polypropylene (PP) microfiber with a millimeter-scale lamellar structure was also fabricated (Figure 2B(v)) [44]. The PP film was cut into lamellar patterns using a laser beam and then placed on a hole-patterned polycarbonate (PC) film with a diameter of 0.6 μm and a length of 20 μm at 145 °C, which is above the melting temperature of PP (130 °C). Subsequently, the melted PP fills the PC film and lamellar structure microhairs are obtained.

Recently, tilted structures have been obtained by applying shearing on a partially cured soft mold with vertical hair structures (Figure 2B(vi,vii)) [45,46]. This method has advantages because shearing distance can determine the tilted angle of the hair structure, unlike structures with a predetermined tilt angle. The fabrication process includes silicon master mold preparation with hair structures prepared via conventional photolithography, creation of a replica of the silicon master using soft materials like PDMS, casting and curing processes; the soft mold should be laterally sheared to tilting before full curing. Figure 2B(vii) shows a PU-based hierarchical tilted structure with a diameter of 20 μm and a length of 60 μm in the first layer, and a diameter of 7 μm and length of 20 μm in the second layer. Here, the soft mold is prepared using PDMS. A partially cured PDMS mold is obtained after curing at 75 °C for 40 min and is laterally pulled for tilting with an angle range from 60° to 90° with shear distance. The tilted soft mold is fully cured at 75 °C for 24 h. Then, PU is poured into the mold and fully cured at 75 °C for 72 h. Tilted prismatic hairs can also be obtained using an identical overall fabrication process, but adopting a prismatic design that activates gripping or releasing and facilitates reusability over 30 cycles (Figure 2B(vii)). The prismatic hair consists of a triangular base (30 μm × 25 μm × 25 μm, and 80 μm in height), and a rectangular tip (20 μm × 7 μm, and 20 μm in height).

Other approaches for fabricating an angled structure regardless of the shape of the master mold exploit nanodrawing methods that utilize the relatively high adhesion between the mold and polymer (Figure 2C,D(i)) [23]. Materials like PS and PMMA are spin-coated on a flat substrate and then a nanohole-patterned master mold is placed on the polymer-coated substrate. After the temperature increases beyond the glass transition temperature, polymers fill molds via capillary force lithography [48,49]. In the process of demolding, the replicated structures stretch due to the adhesive forces between the mold and polymer, where the level of stretching is determined based on the drawing temperature. At this point, the structure becomes tilted because of the demolding direction and the relatively low mechanical modulus of polymers. Polyethylene (PE) microhairs were fabricated (Figure 2D(ii)) using a technique similar to the above drawing methods; the key difference was that the degree of elongation was not controlled by the delaminating temperature, but by the peeling angle. The hair angles decreased with increasing pull angle. Tilted hairs with an average length of 55.7 μm and a radius of 1.17 μm were obtained [47].

### 3.2. Control of the Geometry of Polymeric Nanohairs

#### 3.2.1. Beam Irradiation

**Figure 3:**
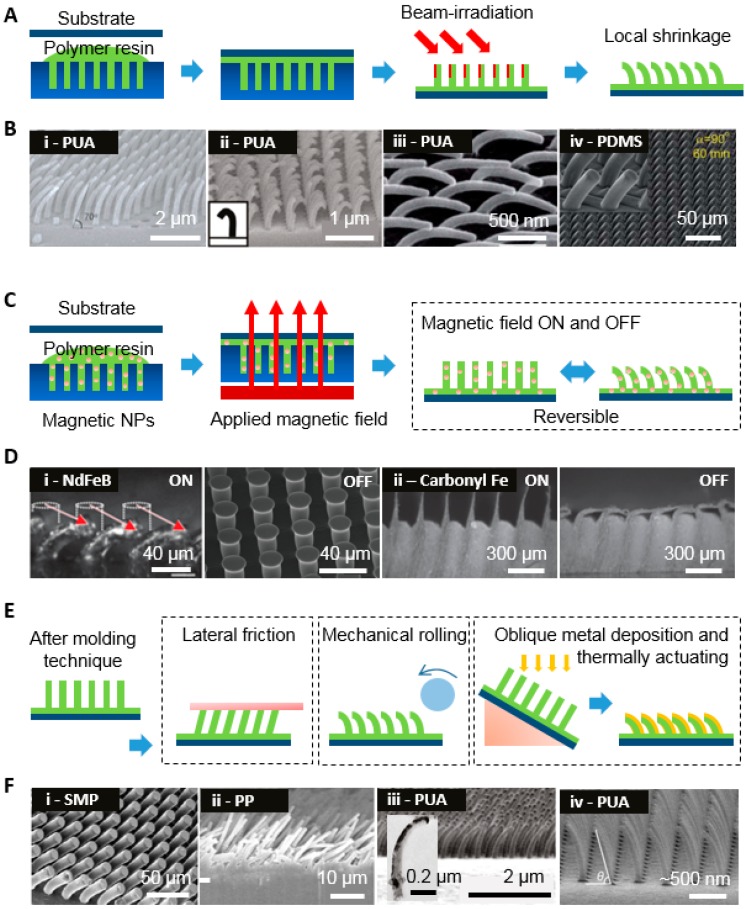
Schematic diagram and representative SEM images of various actuating techniques for fabricating tilted hairs. Vertical micro/nano hairs obtained by molding followed by an additional process to control the geometry of the hairs are shown. (**A**,**B**) Schematic procedures and SEM images for angled e-beam or ion beam irradiation (**A**(i–iii) PUA and **A**(iv) PDMS hairs obtained by e-beam and ion beam irradiation, respectively. **B**(i) reproduced from [26], with permission from John Wiley & Sons. **B**(ii) reproduced from [50], with permission from the American Chemical Society. **B**(iii,iv) reproduced from [51,52], with permission from the Royal Society & Chemistry); (**C**,**D**) Schematic of the procedures and SEM images for manipulating the angle by applying a magnetic field. Tilted PDMS hairs with carbonyl iron or NdFeB obtained by applying a magnetic field (**D**(i,ii) reproduced from [53,54], with permission from John Wiley & Sons); (**E**,**F**) Schematic of the procedures and SEM images of controlling the geometry with shear friction, mechanical pressure by rolling, and anisotropic shrinkage by oblique metal deposition and annealing. **F**(i) Shape memory polymer hairs obtained via lateral friction at elevated temperatures. **F**(ii) PP nanohairs obtained by mechanical rolling. **F**(iii,iv) PUA hairs obtained via oblique metal deposition and thermal annealing (**F**(i) reproduced from [55], with permission from John Wiley & Sons. **F**(ii) reproduced from [56], with permission from AIP publishing. **F**(iii) reproduced from [57], with permission from American Chemical Society. **F**(iv) reproduced from [58], with permission from Elsevier).

In contrast to the previous molding technique, beam irradiation techniques are useful for modulating angle and tip shape (Figure 3A,B). They consist of a two-step process of molding followed by beam irradiation. The replicated patterns from molding are exposed to a post-treatment process of beam actuating, such as e-beam (Figure 3B(i–ii))and ion beam (Figure 3B(iii–iv)) processes [26,50,51,52]. Polyurethane acrylate (PUA) is usually adopted because of its stiffness, which can endure a high aspect ratio (AR > 10) without collapsing, as shown in Figure 3B(i–iii). PUA nanohairs are obtained as vertical structures at first; then, as nanohairs are exposed to the e-beam, angle deformation occurs. This results from the penetration of electrons into irradiated sites, in which the free volume is relatively shrunken. This also reveals that the tilting angle could be modulated depending on the exposure time and beam acceleration voltage. Then, the angle decreases with increasing e-beam exposure time. Crispate hairs are also obtained with a high tilt angle, as shown in Figure 3B(ii). At an angle of 80°, the e-beam is partially exposed to the tip of the hairs that form a crispate structure, whereas at an angle of 30°, a large area of the hair is exposed, so tilted hairs are obtained.

Figure 3B(iii–iv) shows an example of ion beam exposure to PUA and PDMS in order to fabricate a tilted structure. The surface becomes stiff when the ion beam is applied to vertical structures, where wrinkling and shrinkage occur via mechanical mismatch with the other side of the PUA and PDMS, which is not exposed to the beam. In the case of PDMS, the modulus after exposure is about 70 to 100 times that of pristine PDMS (*E* = 1.8 MPa).

#### 3.2.2. Magnetic Field

Recently, magnetically actuated gecko like dry adhesives were reported wherein the structures promptly responded to the application of a magnetic field. In this technique, polymeric elastomers are mixed with magnetic materials such as NdFeB and carbonyl iron particles [53,54]. Then, these mixtures are cast onto a mold and cured by heating in an oven. A magnetic field gradient is consistently applied during curing, which orients magnetic particles in the direction to enhance the magnetic effect. The replicated hair patterns are mounted near the magnet, which facilitates tilting of the hairs.

Figure 3C shows schematic illustrations of magneto-elastomer fabrication with hair shape and directional bending procedure. The representative SEM images with an on and off-mode magnetic field are shown in Figure 3D. Here, the PDMS precursors are blended with NdFeB and then these composites are placed on a microhair-patterned master mold on a magnet that transfers magnetic particles to the end of the hairs [53]. The tilt angle can be modulated by the magnetic field and is also affected by the concentration of NdFeB. In this experiment, NdFeB particles (diameter 6–9 μm) at a concentration of 20% which allows hairs to down to back substrate were used to tilt the vertical structure with a diameter of 25 μm and height of 48 μm. If the concentration is lower than 20%, partially or slightly tilting is available, and higher concentrations do not significantly enhance the hair response due to their massive inclusion. Alternatively, the parallel wedge-shaped microridges spaced 325 μm apart, a tapered tip of 10 μm height, and 15 μm rows shown in the right side of Figure 3E(ii) were fabricated using PDMS casting mixed with carbonyl iron particles (diameter 0.1–1 μm) at a weight ratio of 25% [54].

#### 3.2.3. Mechanical Stress

Both microstructures with low-modulus materials and nanostructures that induce a low effective modulus (via a thin, high-aspect-ratio geometry) are fragile and easily deformed by applied mechanical stress. This method utilizing applied mechanical stress, as shown in the left side of Figure 3E,F(i–iii), is useful for fabricating tilted hairs and it is simple and cost efficient. An example of memory polymer microhairs is shown in the left side of Figure 3E,F(i) [55]. Its initial structure before actuating is obtained via a casting and curing process using arrays with a height of 100 μm and a radius of 10 μm. To actuate the vertical shape into a tilted shape, the glass substrate is placed on the hair structures and subsequently pulled in a parallel direction at the glass transition temperature. Because there is a frictional force between the hair and the substrate, tilted structures are fabricated using a cooling process. The shape memory feature of the polymer is particularly interesting; if the tilted structure is heated again to above the glass transition temperature, it returns to its original form. However, it cannot return deformation status. Therefore, it is expected that the structure will have potential for various applications that require repeated tuning. Figure 3F(ii) illustrates that the angled hair structures consist of polypropylene (PP) [56]. In this technique, vertical hairs are molded from polycarbonate filter and rolling is applied in a certain direction such that a tilted structure with a diameter of 0.6 μm and a length of 18 μm is obtained. In this manner, we could produce directional adhesion.

#### 3.2.4. Thermal Shrinkage

Due to its controllable angular degree, anisotropic thermal shrinkage represents another important class of tilted hair fabrication methods. This approach utilizes the thermal expansion coefficient of various materials as a driving force. In this technique, two fabrication methods have been demonstrated, including one-step or two-step processes. The two-step process is an earlier approach that requires an additional annealing process (Figure 3E) [57]. Vertical nanohairs with a diameter of 100 nm and a height of 900 nm are obtained via cast molding and UV curing of PUA. Then, a thin layer of platinum (Pt) less than 12-nm-thick is obliquely sputter-deposited on a nanohair sample. To obtain the tilted structure, the Pt-coated nanohair samples are heated from 20 to 160 °C for 30 min. Mismatch of thermal coefficients cause vertical hairs to be tilted towards the material with a small coefficient. In addition, the angle decreases with increasing annealing temperature. Secondly, fabrication via the one-step process is nearly identical to fabrication via the two-step process, but varies in terms of metal thickness, type of metal, and the metal coating; thermal evaporation is adopted instead of sputtering [58]. The actuating mechanism also varies. In the two-step process, the thermal coefficient is a crucial factor for determining bending direction, whereas in the one-step process, the tensile or compressive stress that occurs due to the 20–30-nm-thick metal coating during thermal evaporation determines bending direction. The Au-deposited sample is tilted toward the metal face, while the aluminum (Al)-deposited sample is tilted toward the polymer face.

## 4. Limitations of Tilted Nanohairs and New Strategies to Overcome These Limitations

As we discussed in the introduction, Gecko setae, which consist of stiff β-kerotin, have remarkable durability, reliability, and adhesion even on rough surfaces [17]. However, artificially synthesized gecko-like dry adhesives do not satisfy all of the requirements mentioned above. Therefore, we need to determine the limitation of previous research and seek solutions. The current limitations of this technology include: (1) durability of tilted nanohairs against repeated contact; (2) enhancement of adhesion via optimization of the tip surface; and (3) adhesion on a rough surface. The following chapters will describe current approaches to overcome these limitations.

### 4.1. Geometric Effects of the Nanohairs

#### 4.1.1. Tip Shape Effect

**Figure 4:**
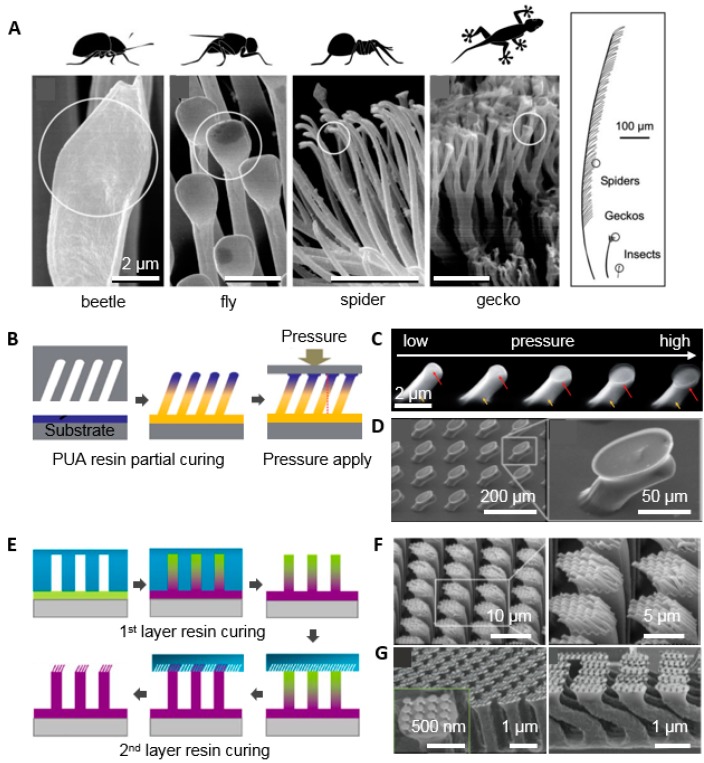
(**A**) Various tip and hierarchical structures for attachment in nature (reproduced from [8], with permission from the National Academy of Science, Washington, DC, USA); (**B**,**E**) Schematic diagrams for fabricating a mushroom tip shape and hierarchical structure by the two-step process (**B**: Reproduced from [67], with permission from the Royal Society & Chemistry; **E**: Reproduced from [41], with permission from National Academy of Science, Washington, DC, USA); (**C**,**D**) Mushroom nanohairs obtained by pressing the tip of the partially cured PUA hair and inked PDMS tip (**C**: Reproduced from [67], with permission from the Royal Society & Chemistry; **D**: Reproduced from [68], with permission from John Wiley & Sons); (**F**,**G**) Hierarchical nanohairs obtained by sequential casting and curing processes (**F**: Reproduced from [41], with permission from the National Academy of Science, Washington, DC, USA; **G**: Reproduced from [69], with permission from the American Chemical Society).

In a variety of organisms with a hairy attachment system, the tip of this structure consists of a paddle-like shape, but there are differences in the sizes of the structures. As body weight increases, the structures are scaled down from microscale to nanoscale (Figure 4A) [8]. In particular, geckos have a finer structure than that of beetles, flies, and spiders. The diameters of the tips range from 200 to 500 nm. In previous work, the adhesion properties by modification of tip geometry from a hemisphere to mushroom structure were amplified over 30 times [59,60,61,62,63,64,65,66,67,68]. These structures could be obtained by additional planarization processes including pressing the tip after replication of tilted hair arrays using the molding technique shown in Figure 4B [67]. As pressure is applied, the hemisphere tip of the tilted hair is gradually deformed into a mushroom structure, which maximizes the contact area. The oxygen between the PUA precursor and the master mold hinders polymerization between the initiator radical and monomers, which results in a partially cured tip and a fully cured body. Therefore, the elastic modulus of the tip is lower than that of the fully cured body. As the applied pressure increases to 5 × 10^4^ Pa, the diameter of the hair tip increases to 1300 nm from the initial 700 nm (Figure 4C). The dipping method shown in Figure 4D also enhances the contact area [68]. Tilted hairs are fabricated via an etched master mold with a certain angle and are subsequently dipped in polyurethanes (PU) that is spin-coated on the substrate with a low surface energy. Then, the angles are changed from 0° to 90° via applied pressure. These microhairs have diameters of 35 μm and lengths of 100 μm.

#### 4.1.2. Hierarchical Structure for High AR Nanostructures

It has been reported that a single layer of nanoscale pillar structures does not intimately contact the target substrate because of microscale roughness, whereas hierarchical structures maintain adhesion on rough surfaces [70,71]. The high AR nanostructure with a tilted angle enables high adhesion even on rough surfaces (which reduce the projected contact area in low AR adhesives), and lowers the effective modulus that make easily each hair deformed to surface [17]. Figure 4E–G illustrates two different scale molding methods and replicated PUA hierarchical structures whose size is based on actual gecko setae and spatulae [37,69]. The first setae-motivated layer with a diameter of 4 μm and length of 25 μm on a 50-μm-thick PET film was obtained by PUA casting and was then located on a second spatula-like mold with a diameter of 350 nm and length of 2.8 μm. Here, the tip of the first layer is partially cured because trapped oxygen scavenges initiator radicals and could be further molded on predefined structures. In this process, the AR increases. The adhesion experiments were performed on rough surfaces with different roughnesses. The results indicated that the hierarchical structure had better adhesion than the monolayer structure because the structure allows each hair to reach deeper areas due to secondary levels of deformation. In the case of monolayer structures, the contact areas are determined by the size and aspect ratio of the initial hair, whereas hierarchical structures cause deformation of relatively more hairs independently at microscale roughness, expanding the contact area and resulting in high adhesion. The hierarchical patterns were also prepared by angled molding and dipping methods were adopted subsequently, as shown in Figure 4G [69]. These patterns are composed of a tilted first layer with a diameter of 400 μm and a vertical height of 2 mm; a second layer of hairs with a height of 100 μm and a diameter of 50 μm; and mushroom tips with a diameter of 100 μm.

### 4.2. Enhanced Durability of Nanohairs

**Figure 5:**
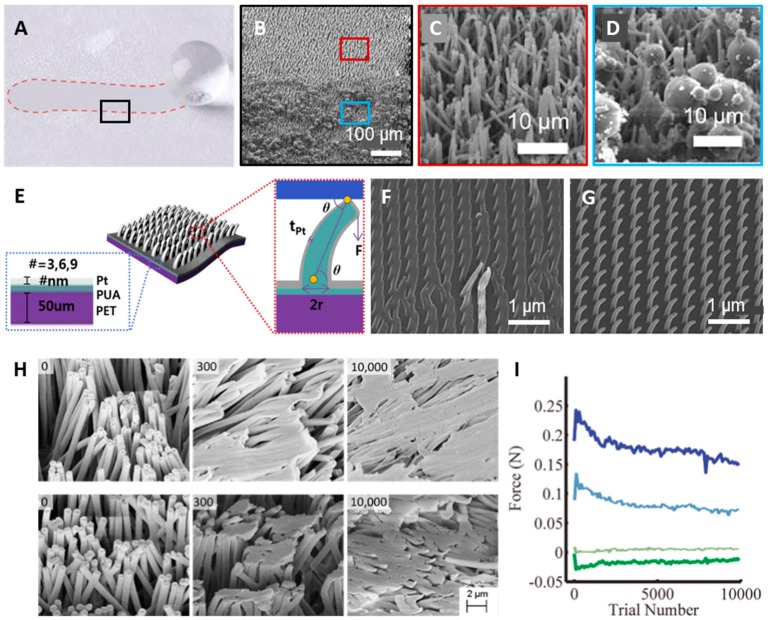
Enhanced durability and repeatability of micro/nanohairs. (**A**) Image of a sliding water droplet on microhair structures contaminated with ceramic microspheres; (**B**) SEM image of the boundary between (**C**) the self-cleaned area and (**D**) the contaminated area (**A**–**D**: Reproduced from [21], with permission from the American Chemical Society); (**E**) Schematic illustration of thin-metal-coated nanohairs; (**F**) SEM image after a 10-cycle adhesion test; and (**G**) Pt-deposited nanohairs after a 100-cycle adhesion test (**E**–**G**: Reproduced from [72], with permission from IOP publishing Ltd.); (**H**) SEM images of HDPE (top) and PP (bottom) after 0, 300, and 10,000 cycles; (**I**) Maximum shear force and normal force of HDPE and PP, respectively (H and I: Reproduced from [25], with permission from the American Chemical Society).

The reversible properties and repeatability of dry adhesives are among their most important characteristics because commercialized pressure sensitive adhesive (PSA), such as that used in 3 M tape, experiences significantly degraded adhesion upon repeated use. A gecko can maintain its adhesion force even after 30,000 attachment tests without significant damage to hair structure [9]. However, synthetic gecko-like dry adhesives have not achieved similar reusability, whereas the adhesion force of artificial micro and nanohairs has matched and even surpassed that of gecko feet. One of big hurdles for reusability is contamination. Contact with dust and particles can eventually cause adhesion degradation. Recently, this has been overcome by adopting relatively hard polymers, such as HDPE, which has a self-cleaning effect with water or gas blowing (Figure 5A–D) [19,20,21,22]. The other hurdle is that low mechanical properties easily lead to nanohair collapse, causing hair-to-hair pairing or hair-to-bottom surface adhesion. Integration of a soft polymer and a stiff metal has emerged as a solution to this, as it increases the effective modulus of the hairs, as illustrated in Figure 5E–G [72]. Nanohairs and their backbone were fabricated with intrinsically soft materials, and their effective moduli can be enhanced by the deposition of metal. The nanohairs with enhanced durability that are obtained via molding techniques and mechanically actuated after metal coating are based on low-modulus PUA (*E* = 19.8 MPa). Then, they are sputtered with relatively high-modulus platinum (*E* = 168 GPa). Figure 5F,G show SEM images of the patterns of pristine nanohairs after 10 cycles and 6-nm Pt-coated nanohairs after 100 cycles of durability testing, respectively. The Pt-coated nanohairs were robust even after 100 tests. Here, the Pt thickness was modulated to 3, 6, and 9 nm for the 100 cycle repetition experiment. As the Pt thickness increased, the flexural rigidity, which is defined as the force required to bend the hair structure, increased; thus, hairs could endure a higher external load. As a result, the adhesion force decreased with increasing thickness. In this technique, the minimum degradation of adhesion was achieved in the 9-nm sample, whereas this sample showed relatively low adhesion force.

Other researches fabricated hair structures with an elastic modulus similar to that of the gecko (*E* = 2 GPa) and scaled up the size [43,73]. Figure 5H shows the structure with a length of 20 μm and a diameter of 600 nm based on polypropylene (PP, *E* = 1.5 GPa) and HDPE (*E* = 0.4 GPa) [25]. In the durability test, the adhesion forces increased in both PP and HDPE over the first 300 cycles, but decreased up to 10,000 cycles, where they maintained adhesion of 54% and 63% for PP and HDPE, respectively (Figure 5I). The increase in adhesion up to 300 cycles resulted from hair tip deformation, which occurred as the hair changed from a cylinder to mushroom shape, expanding the contact area, as discussed in the previous section. However, the hair’s continuous deformation and inter-hair adhesion resulted in a considerable loss of contact area and, therefore, the adhesion force decreased.

## 5. Applications of Tilted Dry Adhesive

Dry adhesives inspired by geckos have a number of advantages. Their remarkably high adhesion hysteresis with high adhesion force and low detached force facilitates many unique applications. Here, we review two applications of transfer printing tools and robotics. With transfer printing, flexible and heterogeneous electronic devices can be assembled without an adhesive layer. A gecko-inspired climbing robot is also possible.

### 5.1. Transfer Printing Tool

Flexible electronics have attracted considerable attention [74,75,76,77]. Their soft geometry allows for a deformable shape that is easily mounted on human skin as wearable technology. Wearable devices for personal health sensing and therapy are a popular research topic. In these devices, conformal contact is a crucial factor because incomplete contact prohibits accurate electrophysiological signal sensing. To achieve intimate contact with the body, flexibility and stretchability are essential, but conventional devices are fundamentally brittle and planar [78]. This approach is not compatible with the curvilinear surfaces of the human body. Also, for flexible electronics, heterogeneous materials and devices should be monolithically integrated. Integration of conventional inorganic semiconductor materials with unconventional materials has recently emerged as a key solution to achieve flexibility and stretchability [79,80,81,82,83].

**Figure 6:**
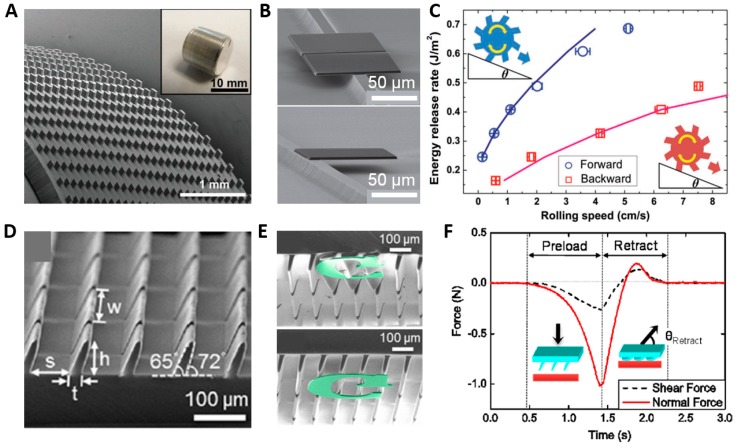

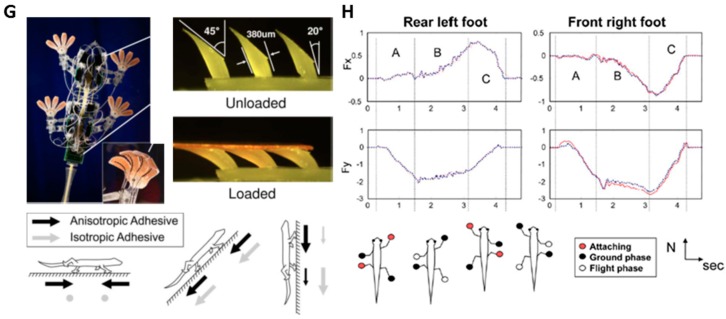
Transfer printing tool and robotics applications. (**A**,**D**) SEM images of angled microstructures for transfer printing (**A**: Reproduced from [4], with permission from John Wiley & Sons; **D**: Reproduced from [5], with permission from the American Chemical Society); (**B**,**E**) Silicon inks transferred on structured adhesiveless substrates (**B**: Reproduced from [4], with permission from John Wiley & Sons; **E**: Reproduced from [5], with permission from the American Chemical Society); (**C**,**F**) The mechanism for the energy release rate with two different directions of the angled posts (**C**: Reproduced from [4], with permission from John Wiley & Sons; **F**: Reproduced from [5], with permission from the American Chemical Society); (**G**) Gecko-like robotics, a Stickybot, with directional PU hairs; (**H**) Adhesion force data of the robot’s feet (**G,H**: Reproduced from [6], with permission from IEEE).

Here, electronic devices are placed on flexible substrates without adhesive layer [74,75]. Picking up and placing processes using directional adhesion (called transfer printing) with PDMS stamp is utilized in assembling of heterogeneous electronic devices even without typical adhesives that have the same adhesion force regardless of attachment and detachment direction [80]. Figure 6A,D show angular PDMS structures (A: lateral dimensions of 100 μm × 100 μm, vertical height of 100 μm, and angle of 17°, D: PDMS, width of 500 μm, root of 25 μm, height of 70 μm, and angle of 72°, respectively) [4,5]. As mentioned, adhesion induced by the angles between an angular structure and plate vary in terms of the gripping direction and opposite direction, respectively. The gripping direction shows relatively higher adhesion than adhesion in the opposite direction, where a different angle results in an energy difference. Figure 6B,C clearly show the result of directional adhesion in which a silicon plate is transferred to a micro ridge structure via an angular stamp. In the case of typical adhesives, if the target is placed in an undesirable position, it is not easy to reposition. Figure 6E shows the transfer printing process. As the as-patterned Si membrane is pressed, the angular microflap collapses, but it shows higher adhesion with a larger contact area than the adhesion between Si and the substrate. Then, a Si membrane is placed at the desired position without an adhesive layer, which is needed in typical adhesives. The adhesive mechanisms are shown in Figure 6C,F with different geometries of the molds.

### 5.2. Climbing Robots

The ability of geckos to climb walls has inspired robot engineers [6,84,85]. Previous works involving climbing robots have utilized suction, magnetics, and adhesives including tape [86,87,88]. These methods have been successful, but the directional structures of gecko-inspired polymeric hairs have the advantages of energy efficiency and self-cleaning such that adhesion is not decreased over time. Promising applications of climbing robots include surveillance and support at disaster scenes. A well-known climbing robot called Stickybot is shown in Figure 6G [6]. It employs a hierarchical structure from the micrometer to the centimeter scale along with directional adhesion. The toes of the Stickybot consist of two types of PU with varying stiffness. A thin polyester fabric is sandwiched between them and directional polymer stalks (DPS) are fabricated by casting and curing of PU (*E* = 300 KPa). DPS was designed as a directional adhesive like gecko’s setae with a tilt angle of 20°, a diameter of 380 μm, a tip angle of 45°, and a thin ~30-μm tip to produce a softer effective stiffness. It has demonstrated the ability to adhere to vertical glass. The force data from the rear left and front right foot are shown in Figure 6H as the Stickybot climbs glass. However, because DPS is a PU-based soft material, the adhesion degrades over time. The DPS needs to be cleaned periodically after climbing.

## 6. Conclusions

The tilted micro/nanostructure of gecko toes shows exceptional adhesion characteristics, such as directionality, adhesion on various rough surfaces, and reusability, which are unique compared to the adhesion mechanisms of insects, other animals, and commercial adhesives. To mimic this geometry, a variety of tilted hair structures have been fabricated, particularly using polymeric materials, to facilitate a wide range of fabrication techniques such as beam irradiation, magnetic, thermal, and mechanical actuating as well as molding techniques. Fabricated synthetic gecko dry adhesives from previous works showed similar or higher adhesion force. In order to achieve an enhanced dry adhesive system, further studies should be conducted for optimization of the tilted hairs’ tip and hierarchical geometry as well as a high aspect ratio, tilted structure, and reusability, which are crucial factors for practical applications.

## Abbreviations

The following abbreviations are used in this manuscript:

PS	polystyrene
HDPE	high-density polyethylene
SU-8	ultraviolet curable epoxy
PUA	polyurethane acrylate
PC	polycarbonate
PDMS	polydimethyl siloxane
PP	polypropylene
PSA	pressure sensitive adhesive
PI	polyimide
CNT	carbon nanotube
PE	polyethylene
Pt	platinum
Al	aluminum
DPS	Directional Polymer Stalks
